# A Rare Case of Neonatal Kaposiform Hemangioendothelioma With Kasabach–Merritt Phenomenon Presenting as an Abdominal Mass Without Cutaneous Manifestations

**DOI:** 10.1155/crh/5825111

**Published:** 2026-06-12

**Authors:** Su Young Park, Amrryn Halari, Jessenia Palacio-Meadows, Nehal S. Parikh, Danielle Bentsen, Surasak Puvabanditsin

**Affiliations:** ^1^ Rutgers Robert Wood Johnson Medical School, New Brunswick, New Jersey, USA, rutgers.edu; ^2^ Rutgers Cancer Institute, New Brunswick, New Jersey, USA

## Abstract

Kaposiform hemangioendothelioma (KHE) is a rare vascular neoplasm that typically presents in infancy or early childhood, classified as a locally aggressive/borderline vascular tumor. It most commonly involves superficial and deep soft tissues, often demonstrating infiltrative growth into adjacent muscle and bone, whereas retroperitoneal or intraabdominal presentations are less frequent. KHE is frequently complicated by Kasabach–Merritt phenomenon (KMP), a severe consumptive coagulopathy characterized by profound thrombocytopenia, microangiopathic hemolytic anemia, and hypofibrinogenemia with life‐threatening sequelae. We report a case of a male neonate who developed KMP secondary to an abdominal KHE without overlying cutaneous involvement. Diagnosis was established through integration of clinical features, imaging findings, hematological evaluation, and confirmatory biopsy. This patient was managed with a multimodal regimen of sirolimus, corticosteroids, and vincristine, resulting in significant clinical improvement. This case underscores both the diagnostic complexity and therapeutic challenges of KHE complicated by KMP, particularly when lesions occur in deep anatomical sites. Given the rarity of this condition, current management strategies rely largely on expert consensus and case‐based experience, though recent randomized trial and consensus have established sirolimus‐based regimens as highly effective first‐line therapy.

## 1. Introduction

Kaposiform hemangioendothelioma (KHE) is a rare vascular neoplasm with an estimated prevalence of approximately 0.91 per 100,000 children [1]. The etiology of KHE remains unclear. Approximately half of cutaneous cases are detectable at birth, and up to 90% become clinically evident within the first year of life [2]. Clinically, KHE most commonly presents as a solitary lesion, ranging from an erythematous papule to a violaceous indurated mass [3], or as a locally aggressive cutaneous blue–purple tumor [4]. Visible cutaneous lesions are present in approximately 91% of cases, with edema or swelling in 44% [5]. Lesions most frequently arise on the extremities—particularly over joints—with the lower extremities being the most common site (35%), followed by the trunk (29%), the head and neck region (24%), and the upper extremities (10%) [1, 4]. KHE has a propensity to infiltrate deeply into adjacent soft tissues and, in some cases, extend into underlying bone, with destructive changes or remodeling of adjacent bone observed in 42% of cases [6, 7]. Larger lesions (> 5 cm) are associated with an increased risk of Kasabach–Merritt phenomenon (KMP) [8]. Morphologically, KHE can be categorized as superficial (confined to skin and subcutaneous tissue), mixed (involving both superficial and deep structures), or deep (restricted to deep muscle, bone, or joints without cutaneous manifestations). The mixed type is most common, accounting for approximately 63% of cases [8], while approximately 11% of patients lack cutaneous findings [1]. Noncutaneous KHEs may arise in the bone, mediastinum, retroperitoneum, or intraabdominal sites and present significant diagnostic challenges due to their concealed location and absence of visible clinical signs.

KHE poses significant risks of serious complications and morbidity due to its locally aggressive growth, potential to compress adjacent structures, and possibility of developing life‐threatening consumptive coagulopathy. The most critical complication is KMP, a coagulopathy characterized by severe thrombocytopenia (frequently below 50 × 10^9^/L), microangiopathic hemolytic anemia, hypofibrinogenemia, increased fibrin degradation products, and elevated risk of both bleeding and thrombosis. KMP is now recognized to occur exclusively in association with KHE and tufted angioma (TA), which are considered part of the same neoplastic spectrum [9, 10]. Approximately 60%–78% of patients with KHE develop KMP with pathophysiology involving platelet trapping and activation within the abnormal tumor vasculature, resulting in systemic coagulopathy [1, 4, 8, 11]. In severe cases, mortality rates have historically ranged from 20% to 50%, with fatal outcomes attributed to hemorrhage, disseminated intravascular coagulation, and progressive organ dysfunction [10, 12], though modern treatment series report substantially lower mortality [13].

The diagnosis of KHE typically requires an integration of clinical presentation, imaging findings, and hematological abnormalities, with histological confirmation when feasible [14]. Magnetic resonance imaging (MRI) is the preferred modality for lesion characterization, given its superior soft tissue resolution and ability to delineate the extent of infiltration [5, 7, 15]. Characteristic MRI features include heterogeneous hyperintensity on T2‐weighted sequences, ill‐defined borders, and intense heterogeneous enhancement [5, 7]. A biopsy may be contraindicated in cases of severe thrombocytopenia due to the potential for worsening coagulopathy [11, 16].

Surgical excision, while potentially curative, is often unattainable due to the extent of the lesion and its infiltrative growth pattern [17]. Consequently, the management of KHE, both with and without KMP, is primarily medical. Sirolimus (rapamycin), an mTOR inhibitor, has emerged as a highly effective therapeutic option with a multicenter study and a systematic review demonstrating positive outcomes in over 96% of patients, including improvement in tumor size, symptoms, and laboratory parameters [2, 18]. A landmark randomized clinical trial demonstrated that sirolimus plus prednisolone achieved a durable platelet response (> 100 × 10^9^/L) at Week 4 in 94.6% of patients compared to 66.7% receiving sirolimus monotherapy, establishing combination therapy as a valid first‐line treatment for KHE with KMP [19]. Additional systemic treatments include vincristine and corticosteroids, with recent multicenter data showing overall treatment response rates exceeding 70% at 6 months regardless of agent used [13].

Here, we report a male neonate with abdominal KHE complicated by KMP, successfully managed with sirolimus, adjunctively with corticosteroids and vincristine. This report highlights the diagnostic and therapeutic challenges of this uncommon but potentially fatal condition in the neonatal population.

## 2. Case

An early‐term male infant was born at 37 weeks and 6 days of gestation to a 22‐year‐old Hispanic G2P1001 mother via normal spontaneous vaginal delivery at a community hospital with a Level II NICU. Maternal history was notable for a prior first‐trimester miscarriage and hyperemesis gravidarum. Prenatal laboratory testing was negative, and antenatal imaging was limited to a single second‐trimester ultrasound reportedly demonstrating normal anatomy. Rupture of membranes lasted 10 h, and the amniotic fluid was meconium‐stained.

The infant’s Apgar scores were 8 and 9 at 1 and 5 min, respectively. Birth weight was 2693 g (8th percentile), length was 49.5 cm, head circumference was 33 cm (12th percentile), and abdominal girth was 29.5 cm. The initial physical examination at nursery admission was unremarkable. The nursery course was uncomplicated, with the infant tolerating formula feeds without difficulty. Circumcision was performed on Day 2 of life at parental request without immediate postoperative complications. However, on the following day, a 1.0 × 0.5 cm penile hematoma with persistent oozing was observed on the ventral aspect of the penis just below the glans, with two punctate areas of slow bleeding. Two applications of silver nitrate failed to achieve hemostasis.

Due to ongoing bleeding, laboratory evaluation revealed severe thrombocytopenia with a platelet count of 6000/μL, declining to 4000/μL within two hours, prompting immediate platelet transfusion. This infant was also anemic, with a hemoglobin and a hematocrit of 9.4 g/dL and a hematocrit of 27.4%, and a reticulocyte count of 7.02%. Phototherapy was initiated for indirect hyperbilirubinemia. G6PD levels were within normal limits. Coagulation studies showed a prothrombin time (PT) of 15.5 s, a partial thromboplastin time (PTT) of 45.6 s, and an international normalized ratio (INR) of 1.34. The infant appeared lethargic and less active. Given concern for infection, a blood culture was obtained, and empiric antibiotic therapy with ampicillin and gentamicin was initiated.

By Day 3 of life, the infant was transferred to a Level III NICU for further evaluation. On admission, vital signs were as follows: heart rate 156 beats per minute, respiratory rate 42 breaths per minute, blood pressure 71/39 mmHg, temperature 36.9°C, and oxygen saturation of 99% on room air. Physical examination revealed an ill‐appearing infant with a soft cry. The abdomen was soft, full, and nontender, with the liver palpable 2 cm below the costal margin. Genitourinary examination showed normal male genitalia with cauterized glans postcircumcision and no active bleeding. Capillary refill was delayed to 4 seconds. The skin examination revealed oozing at the intravenous insertion site on the left arm and a hematoma surrounding the intravenous site at the right antecubital fossa; the infant appeared pale. Neurologic examination demonstrated normal tone and strength, with intact primitive reflexes.

Despite platelet transfusion prior to transfer, the response was transient; posttransfusion platelet counts rose to 46,000/μL, but rapidly declined to 12,000/μL within 12 h. The patient also received a packed red blood cell (pRBC) transfusion for severe anemia (hemoglobin 5.7 g/dL, hematocrit 16.9% on admission). Following umbilical central line placement, an abdominal radiograph confirmed appropriate catheter position and demonstrated mild gastric distension without other obvious abnormalities.

Pediatric hematology and oncology were consulted. Given the clinical presentation, neonatal alloimmune thrombocytopenia (NAIT) was initially suspected. Intravenous immunoglobulin (IVIG) at 1 g/kg/day was initiated, and platelet transfusion was recommended if counts remained below 50,000/μL in the absence of active bleeding, to mitigate the risk of intracranial hemorrhage. Human platelet antigen (HPA) genotyping was obtained for both parents and the infant, and crossmatched platelets compatible with maternal plasma were sought.

A systemic evaluation was undertaken to refine the differential diagnosis. Maternal platelet counts during pregnancy were within normal limits, rendering autoimmune thrombocytopenia unlikely. There was no evidence of perinatal asphyxia or placental insufficiency, making a transient nonimmune consumptive process unlikely. The infant was nondysmorphic, and the peripheral smear showed no abnormal platelet morphology. Neonatal malignancies, including neuroblastoma, hepatoblastoma, vascular tumors, and, less commonly, sarcomas, were also considered. Tumor marker evaluation demonstrated a markedly elevated alpha‐fetoprotein (AFP) of 33,995 ng/mL, which declined to 2384 ng/mL, consistent with the expected neonatal physiologic downward trajectory. Carcinoembryonic antigen (CEA) was 2.8 ng/mL, and human chorionic gonadotropin (hCG) was less than 1 mIU/mL. Urinary catecholamine metabolites were within normal reference ranges (homovanillic acid [HVA], 15.0 × 10^2^ mg/d; reference range: 3.0–21.0 × 10^2^ mg/d; vanillylmandelic acid [VMA] 11.1 × 10^3^ mg/d; reference range: 3.0–17.0 × 10^3^ mg/d). In the absence of elevated catecholamines, neuroblastoma was unlikely.

Despite repeated platelet transfusions—including PLA antigen–negative units when available while awaiting confirmatory NAIT studies and crossmatching—and a 3‐day course of IVIG (1 g/kg/day), platelet count responses were minimal, with posttransfusion counts peaking just above 50,000/μL and rapidly declining. Consequently, the infant required multiple platelet transfusions daily, up to three per day. Anemia also persisted, necessitating multiple pRBC transfusions during the hospitalization. Coagulation studies were mildly abnormal (PTT: 33.0–35.9 s; PT: 11–15 s; INR: 0.97–1.32). The test for NAIT subsequently returned negative.

**FIGURE 1:**
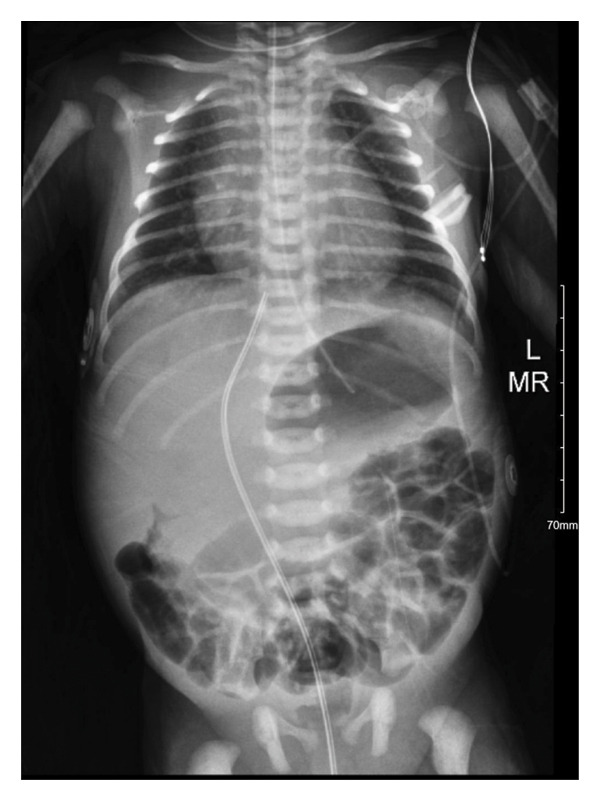
Supine chest–abdomen radiograph shows a markedly distended abdomen with relative paucity of bowel gas in the upper abdomen, most prominent near the hepatic margin, and inferior displacement of gas‐filled small‐bowel loops into the lower quadrants.

**FIGURE 2:**
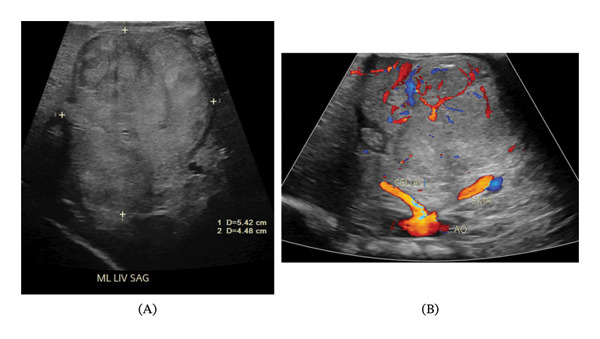
(A) Gray scale ultrasound images demonstrate a 5.4 × 4.5 cm heterogeneous solid mass centered in the mesentery of the abdomen and inferior to the pancreas. (B) Midline sagittal color ultrasound images demonstrate the mass is anterior to the aorta, celiac axis, and superior mesenteric artery. It is hypervascular and supplied by both the celiac artery and the superior mesenteric artery.

At 1 week of age, the infant developed progressive abdominal distension, nonbilious emesis, and tarry stools. Fecal occult blood testing was positive. Coagulation studies revealed hypofibrinogenemia (53 mg/dL), and given symptomatic bleeding with low fibrinogen, the infant was transfused with fresh frozen plasma (FFP). Persistent abdominal distension and concern for gastrointestinal bleeding prompted repeat abdominal radiography, which demonstrated a paucity of bowel gas in the upper quadrant along the hepatic margin with inferior displacement of the bowel loops, raising suspicion for an intraabdominal mass (Figure 1). Targeted abdominal ultrasonography subsequently revealed a heterogeneous, hypervascular solid mass measuring 5.4 × 4.5 cm with internal calcifications, situated in the mid‐abdomen inferior to the pancreas and anterior to the celiac axis and superior mesenteric artery (Figure 2).

The identification of an intraabdominal mass with calcifications in a neonate with severe thrombocytopenia and coagulopathy prompted consideration of a broad differential diagnosis. Necrotizing enterocolitis (NEC) was considered, given the overlapping features of abdominal distension, feeding intolerance, bloody stools, and thrombocytopenia. However, thrombocytopenia preceded the gastrointestinal symptoms by several days, and abdominal radiography did not demonstrate pneumatosis intestinalis, portal venous gas, or pneumoperitoneum. The ultrasonographic finding of a discrete hypervascular solid mass was also inconsistent with NEC. Meconium peritonitis was also entertained, as it is a recognized cause of neonatal abdominal calcification; however, meconium peritonitis characteristically produces linear, plaque‐like peritoneal calcifications along serosal surfaces, often with ascites or pseudocyst formation—none of which were present. Infectious etiologies, including congenital TORCH infections, were also evaluated; however, these conditions are typically associated with systemic findings such as intrauterine growth restriction, purpuric skin lesions, hepatosplenomegaly, or abnormal neuroimaging. None of these features was present. The head ultrasound was normal, and the calcifications were localized within a discrete mass rather than distributed diffusely. Neuroblastoma, the most common malignant abdominal neoplasm in neonates, remained an important consideration given the frequent presence of calcifications. However, urinary catecholamine metabolites (HVA and VMA) were within normal reference ranges, and the mass was mesenteric rather than adrenal or paraspinal in location, reinforcing that this diagnosis was unlikely.

**FIGURE 3:**
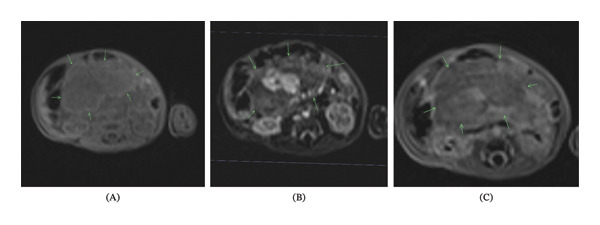
(A) An axial T1‐weighted image through the mass demonstrates a hypointense to isointense mass lesion. (B) Postgadolinium T1‐weighted subtraction images obtained during the arterial phase of imaging, demonstrating heterogeneous enhancement of the lesion with a large central area of intense enhancement. Also, tubular enhancement in the periphery is consistent with arterial enhancement. (C) Postgadolinium T1 5 min delayed subtraction images demonstrate heterogeneous enhancement throughout the lesion.

**FIGURE 4:**
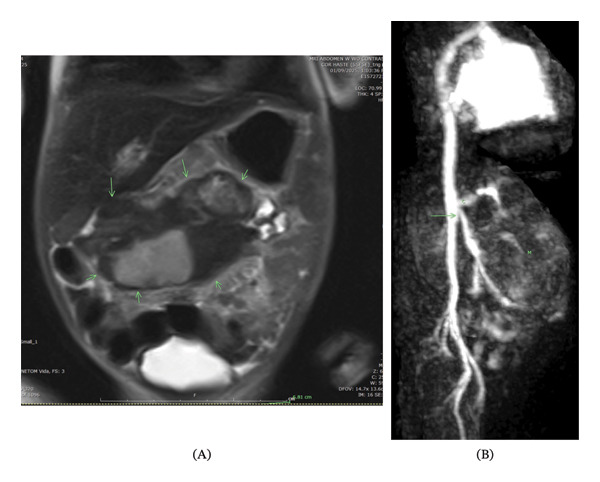
(A) Coronal T2‐weighted MRI images demonstrate a heterogeneous mass lesion centered in the mesentery, which corresponds to the ultrasound findings. (B) MRA images demonstrate that the mass (M) is perfused by the superior mesenteric artery (arrow) and the celiac axis (C).

With these entities excluded on the basis of clinical, laboratory, and imaging findings, the differential diagnosis was narrowed to neonatal vascular neoplasms—infantile hemangioma (IH), KHE, TA, or Kaposiform lymphangiomatosis (KLA). Pediatric surgery was consulted; owing to profound thrombocytopenia and coagulopathy with high hemorrhagic risk, tissue biopsy was deferred, and advanced vascular imaging was recommended. An MRI obtained the same day showed a large mesenteric vascular mass with arterial‐phase enhancement, appearing separate from the liver and adrenal glands and exerting a mass effect on adjacent structures (Figures 3 and 4). Magnetic resonance angiography and venography (MRA/MRV) confirmed a solid mid‐mesenteric lesion supplied predominantly by the superior mesenteric artery (Figure 4). Given the constellation of profound thrombocytopenia, anemia, coagulopathy, and a large infiltrative vascular lesion, KHE with KMP was strongly suspected.

Empiric medical therapy was initiated prior to histologic confirmation due to progressive consumptive coagulopathy and high bleeding risk. Sirolimus was started at 2 weeks of age at an initial dose of 0.8 mg/m^2^ per dose twice daily (0.15 mg/dose). After 1 week, the dose was uptitrated to 0.2 mg/dose, achieving a therapeutic trough level of 10.8 ng/mL. Adjunctive corticosteroid therapy was started with a methylprednisolone loading dose, followed by maintenance dosing at 2 mg/kg/day and subsequently tapered to 1.5 mg/kg/day. Serial ultrasonography over the subsequent 2 weeks demonstrated no appreciable interval reduction in tumor size; however, hematologic parameters showed gradual improvement, including stabilization of fibrinogen levels and progressive recovery of platelet counts, supporting treatment response. The improvement in coagulopathy reduced procedural risk and facilitated a lower‐risk tissue biopsy. The patient was maintained on sirolimus and corticosteroid, with vincristine later added to the treatment regimen.

Prolonged total parenteral nutrition (TPN) was required as persistent feeding intolerance precluded sustained enteral advancement, despite repeated trials. Eventually, as feeding tolerance gradually improved and adequate oral or nasogastric intake was established, TPN was discontinued at approximately 2 months of age. He was discharged at 2.5 months with a weight of 3.385 kg. The nasogastric tube was removed 1 week later when he consistently took 65 mL of fortified formula by mouth. He was followed in the hematology clinic but was readmitted for failure to thrive. However, during this hospitalization, nutritional support was optimized, resulting in catch‐up growth and appropriate weight gain.

During this admission, after approximately 6 weeks of sirolimus therapy, follow‐up MRI/MRA demonstrated a persistent large vascular mass with an interval reduction in size to 4.2 × 5.4 × 4.6 cm. Given adequate weight gain and interval tumor reduction, the decision was made to proceed with a biopsy. An ultrasound‐guided biopsy under general anesthesia was performed at 3 months of age. The biopsy revealed spindled endothelial cells forming slit‐like spaces and small capillaries in a background of fibrous stroma. Occasional rounded nodules of endothelial cells were present throughout the lesion. There was a prominent hemosiderin pigment deposition. Occasional larger thin‐walled vascular spaces and small veins were also present. Cytologic atypia was not identified. Rare mitotic figures were present; however, atypical mitotic figures were not seen. CD31 showed diffuse endothelial cell staining. D2‐40 (podoplanin) highlighted a subset of the spindled cells, with absent staining within the rounded nodules of indeterminate significance. Taken together, the morphologic and immunophenotypic features supported a diagnosis of KHE.

The procedure was complicated by hemorrhagic shock and cardiopulmonary arrest, requiring resuscitation, endotracheal intubation, transfer to the pediatric intensive care unit (PICU), and stabilization with massive blood product transfusion and sodium bicarbonate administration. A subsequent computed tomography (CT) scan revealed active extravasation along the anteroinferior margin of the mass. Following hemodynamic stabilization and multidisciplinary consultation with interventional radiology and general surgery, operative intervention was deferred. The infant was ultimately discharged on sirolimus monotherapy; vincristine was discontinued after a total of 10 doses (6 weekly, followed by 4 biweekly) once platelet counts normalized. Corticosteroids were gradually tapered after 5 months of therapy; during the taper, the infant developed iatrogenic adrenal insufficiency; therefore, a very slow taper was recommended to avoid complications from abrupt withdrawal. At the 7‐month follow‐up, abdominal ultrasonography showed a stable, hypervascular soft‐tissue mass centered within the mesentery, measuring 5.5 × 2.7 × 4.9 cm.

The infant has since remained clinically stable and continues on sirolimus 0.8 mg twice daily, with a therapeutic trough goal of 10–12 ng/mL. Hematology recommended rTAG genetic testing for specific platelet‐related genes to evaluate for congenital thrombocytopenia syndromes; results were negative. Additional studies—thrombin time; Factors VIII, IX, and XI; and von Willebrand factor antigen and activity—were advised and remained pending at discharge. Hematology will continue to follow the patient as an outpatient, with plans to review these results and consider additional infectious disease testing for TORCH infections.

## 3. Discussion

KHE is a rare congenital vascular neoplasm of endothelial origin that histologically resembles Kaposi’s sarcoma (KS) [17]. The exact incidence of KHE remains unclear, although one large medical center–based study estimated the prevalence at approximately 0.91 cases per 100,000 children [1] KHE most often presents during infancy and the first decade of life without clear gender predilection; in one cohort of 146 patients, 91.8% of lesions developed within the first year of life, with a median age at diagnosis of 2.3 months [8]. KHE is characterized by locally aggressive and infiltrative growth, often extending into deep tissues and across fascial planes [20]. It was first delineated as a distinct entity by Zukerberg et al. in 1993 [21]. Although KHE is now generally considered a distinct entity from other vascular neoplasms—such as Kaposi‐like hemangioma, hemangioma with Kaposi‐like features, Kaposi‐like infantile hemangioendothelioma, and congenital hemangioendothelioma—its rarity has historically hindered comprehensive characterization of its immunophenotypic profile and limited understanding of its long‐term clinical behavior [22].

KHE demonstrates distinctive histopathological features that allow differentiation from its main differentials, such as IH and KS. As seen in the biopsy from our case, the tumor is composed of infiltrating, lobulated nodules with slit‐like or crescentic vascular channels that extend into various tissue planes, including the subcutaneous fascia and muscular layers. These poorly canalized vessels are lined by spindle‐shaped endothelial cells, which often form focal lumina containing trapped red blood cells [23]. Associated findings include platelet thrombi, eosinophilic hyaline bodies, and hemosiderin deposition—features that reflect localized red cell destruction and are uncommon in both IH and KS [21, 23].

Immunohistochemistry further aids in establishing a definitive diagnosis. Glucose transporter 1 (GLUT1) is strongly expressed in IH, particularly in the cellular phase, but is consistently negative in KHE [22]. In contrast, KHE demonstrates epithelioid or glomeruloid foci that show immunoreactivity for CD31, CD34, and Friend leukemia integration 1 (FLI1) transcription factor, with prominent smooth muscle actin (SMA), positive cells, suggestive of pericytic involvement [22]. On the other hand, KS is pathogenetically linked to Human herpesvirus‐8 (HHV‐8) transcripts, which are not detected in KHE [22, 24]. D2‐40 immunohistochemistry is a useful marker for KHE, staining the neoplastic spindled cells and lymphatic channels, and is particularly helpful in differentiating KHE from IH, congenital hemangiomas, and pyogenic granuloma, none of which immunoreact with D2‐40 [25].

Based on morphologic characteristics, KHE can be classified into three categories: superficial (confined to the skin and subcutaneous tissue without involvement of deeper structures), mixed (involving both superficial structures and deep tissues such as muscle, bone, or joints), and deep (restricted to deep muscle, bone, or joints without cutaneous manifestations). The mixed subtype is the most common, accounting for approximately 63% of cases [2]. In a cohort of 107 patients, Croteau et al. [1] reported that only 11% of patients lacked cutaneous findings, with cutaneous KHE favoring the extremities, especially overlying joints. These noncutaneous lesions, frequently retroperitoneal, intraabdominal, or deep within the intramuscular tissue, present diagnostic challenges due to their concealed location and absence of visible clinical signs [1].

Retroperitoneal or intraabdominal KHE is particularly challenging to diagnose due to its diverse clinical manifestations, nonspecific symptoms, and inconclusive imaging features. Consequently, both diagnosis and initiation of appropriate management are often delayed; in one cohort, the diagnosis of KHE was delayed by ≥ 1 month in 65.7% of patients with KMP [8]. Our patient exemplifies this diagnostic challenge: he presented initially with persistent postprocedural bleeding, severe transfusion‐refractory thrombocytopenia, and anemia, but lacked the typical cutaneous lesions that frequently raise suspicion for KHE. The initial abdominal radiograph did not demonstrate any obvious abnormalities, with subtle findings that were not appreciated at the time of interpretation. Definitive diagnosis was delayed until clinical progression—manifested by increasing abdominal distension and feeding intolerance—prompted recognition of radiographic features suggestive of an abdominal mass, followed by advanced imaging and confirmatory biopsy. Recent expert consensus guidelines underscore the importance of maintaining a high index of suspicion for deep‐seated vascular tumors in infants presenting with unexplained consumptive coagulopathy, even in the absence of cutaneous manifestations [14]. A diagnosis of KHE requires integration of clinical, imaging, and histologic features [14, 16].

Since Zukerberg et al. [21] highlighted the close association between KHE, KMP, and lymphatic abnormalities when the study was first reported, KHE is now recognized as one of the principal vascular tumors associated with KMP in infants and young children. KHE is frequently complicated by KMP; Ji et al. [8] described 146 patients with KHE, and approximately 71% developed KMP [2]. KMP is a severe consumptive coagulopathy characterized by profound thrombocytopenia, microangiopathic hemolytic anemia, hypofibrinogenemia, and depletion of coagulation factors, leading to rapid onset, fulminant progression, and high mortality [26, 27]. Critically, KMP is now recognized to occur exclusively in association with KHE or TA, not with common IH [9, 10]. Underlying pathophysiology involves platelet trapping and activation within the abnormal vasculature of the tumor, resulting in systemic coagulopathy and high risk of bleeding, thrombosis, and multiorgan dysfunction [20, 27].

Tumor size and location are critical determinants of KMP risk. Deep, infiltrative lesions involving the trunk, abdomen, or retroperitoneum carry substantially higher risk [1, 8]. In Ji et al.′s [2] series, young age (< 6 months), large lesion size (> 5.0 cm), and mixed lesion type were significant predictors of KMP in multivariate analysis [2]. Retroperitoneal and intrathoracic lesions were complicated by KMP in 85% and 100% of cases, respectively; compared to superficial lesions, KHE infiltrating into muscle or deeper was 6.3‐fold more likely to manifest KMP and 18‐fold higher if retroperitoneal or intrathoracic [1]. Fatal outcomes were attributed to hemorrhage, high‐output cardiac failure, and organ dysfunction due to infiltrative growth [10, 12]. The size and intraabdominal location of our patient’s lesion placed him at high risk of KMP, which manifested clinically with severe thrombocytopenia refractory to platelet transfusions, anemia requiring repeated pRBC transfusions, and hypofibrinogenemia necessitating plasma replacement.

When feasible and safe, a tissue biopsy remains the diagnostic gold standard; however, in our patient with significant coagulopathy, biopsy was appropriately deferred until hematologic stability was achieved to minimize procedural risk. Contemporary expert consensus algorithms acknowledge that diagnosis may initially rely on clinical presentation and advanced imaging when bleeding risk precludes immediate histologic confirmation [11, 14, 16]. Accordingly, medical treatment was initiated based on clinical presentation and characteristic imaging findings, prior to histologic confirmation, due to the severity of coagulopathy. Subsequent clinical improvement, including stabilization of coagulopathy, allowed delayed biopsy; the diagnosis was ultimately confirmed through a biopsy performed at 3 months of age. Nonetheless, imaging —particularly MRI and MRA — proved invaluable in characterizing the vascular nature of the lesion and its relationship to mesenteric vessels. Because KHE complicated with KMP is rare, evidence‐based treatment guidelines are lacking, and most current recommendations are derived from retrospective studies, case reports, and expert consensus. A multidisciplinary approach, early recognition, histopathologic confirmation when safe, and prompt initiation of targeted therapy remain the cornerstones of management [20]. Surgical excision is rarely feasible because of the tumor’s size, infiltrative nature, and location; therefore, management of KHE, with or without KMP, is predominantly medical [17].

Consensus‐based practice guidelines for the pharmacologic management of KHE focus on reducing tumor size and correcting coagulopathy. Prior to the demonstrated efficacy of sirolimus, systemic corticosteroids and vincristine were the primary treatment options. However, the effects of systemic corticosteroids can vary among patients, with responses often demonstrating inconsistency; a meta‐analysis found a pooled response rate of only 0.27 (95% CI: 0.17–0.36) for systemic corticosteroids compared with 0.72 (95% CI: 0.64–0.79) for vincristine [28]. In addition, prolonged systemic corticosteroid therapy is well known to be associated with adverse effects, including transient growth deceleration, increased susceptibility to infection, and behavioral changes. Vincristine has demonstrated efficacy, particularly in steroid‐refractory cases; in one retrospective study of 37 patients with steroid‐resistant KHE, 26 achieved complete remission with platelet counts reaching normal levels within 7.6 ± 5.2 weeks after vincristine treatment [29]. However, vincristine therapy is associated with certain adverse effects, including peripheral sensory or motor neuropathy [4, 30], vasospastic attacks such as Raynaud’s phenomenon [28, 30, 31], gastrointestinal toxicities including abdominal pain and loss of appetite (FDA Vincristine Label, 2026), and transient hepatic transaminase elevations [32–34].

Sirolimus (rapamycin), a mechanistic target of rapamycin (mTOR) inhibitor, has emerged as a highly effective agent for KHE due to its antiangiogenic and immunomodulatory properties, reducing endothelial cell proliferation and angiogenesis, thereby stabilizing or shrinking vascular lesions [20, 35]. In a multicenter retrospective cohort of 52 patients, Ji et al. [8] reported that 96% and 98% of patients demonstrated improved symptoms and/or complications at 6 and 12 months after sirolimus treatment, respectively. Similarly, a recent multicenter cohort study of 159 patients with KHE/TA demonstrated overall treatment response rates exceeding 70% at 6 months, with comparable efficacy between sirolimus and vincristine, supporting individualized treatment decisions based on clinical context and patient/physician preferences [13].

In cases of severe KMP, combination therapy is frequently utilized. A landmark randomized clinical trial demonstrated that sirolimus plus prednisolone achieved a durable platelet response (> 100 × 10^9^/L) at Week 4 in 94.6% of patients compared to 66.7% receiving sirolimus monotherapy (difference 27.9%; 95% CI: 10.0–44.7) [19]. Patients receiving combination therapy had fewer blood transfusions and a lower total incidence of disease sequelae than patients receiving sirolimus alone [19]. Long‐term follow‐up over a median of 56 months (167 patients) demonstrated sustained efficacy of sirolimus, with 92.2% of patients maintaining a durable response and no evidence of unacceptable cumulative toxicity. However, challenges remained, including treatment nonresponse, tumor relapse following discontinuation (17.3%), and long‐term sequelae [36]. As demonstrated in our case, the patient demonstrated a favorable response and has maintained a stable tumor size on sirolimus in combination with adjunctive corticosteroid and vincristine therapy [37, 38].

This case highlights the diagnostic and therapeutic challenges of intraabdominal KHE in the neonatal period, particularly in the absence of cutaneous manifestations when complicated by the KMP. The lack of visible skin findings may delay recognition, underscoring the need for heightened clinical suspicion in neonates presenting with unexplained intraabdominal masses and coagulopathy. Early diagnosis, supported by imaging and histopathologic confirmation when feasible, along with prompt initiation of targeted medical therapy, particularly sirolimus‐based regimens within a multidisciplinary care framework, is essential to improve outcomes in these high‐risk patients.

## Author Contributions

S.Y.P., J.P‐M., and N.S.P. contributed to data gathering, patient diagnosis, and treatment.

All authors contributed to drafting, writing, and revision of the main manuscript.

## Funding

No funding was received for this study.

## Disclosure

All authors have read and approved the final version of the manuscript, had full access to all of the data in this study, and take complete responsibility for the integrity of the data and the accuracy of the data analysis.

## Ethics Statement

This report did not require approval by our institute’s committee on human research.

## Consent

All the patients allowed personal data processing, and informed consent was obtained from all individual participants included in the study, including written consent of the patient’s parents.

## Conflicts of Interest

The authors declare no conflicts of interest.

## Data Availability

The data supporting this report’s findings are available from the corresponding author upon reasonable request.
